# An unexpected retroperitoneal paraganglioma with hypertensive crisis during surgical resection: a case report and literature review

**DOI:** 10.3389/fonc.2025.1614545

**Published:** 2025-08-01

**Authors:** Meng-Ting Shen, Ye Xuan, Kai-Yu Chen, Xin-Lei Lu, Wei-Ping Lei, Ya-Qin Huang, Jian-Liang Sun

**Affiliations:** ^1^ The Fourth School of Clinical Medicine, Zhejiang Chinese Medical University, Hangzhou First People’s Hospital, Hangzhou, China; ^2^ Department of Anesthesiology, Affiliated Hangzhou First People’s Hospital, School of Medicine, Westlake University, Hangzhou, China

**Keywords:** anesthesia, retroperitoneal paraganglioma, hypertensive crisis, catecholamine, intraoperative management

## Abstract

Retroperitoneal paraganglioma is an extremely rare tumor. Its atypical clinical presentations often lead to missed and misdiagnosis. Here, we report a 60-year-old male with unexplained weight loss and a medical history of hypertension and diabetes. Preoperative evaluations showed a left lower abdominal mass, considered as a malignant gastrointestinal stromal tumor or a leiomyosarcoma. During the laparoscopic resection of tumor, his blood pressure fluctuated significantly, especially with the tumor manipulation that caused hypertensive crisis. Phentolamine infusion was given to control the blood pressure, and the tumor was removed under the open abdominal surgery. Further laboratory tests on catecholamine levels and postoperative histopathology confirmed the diagnosis of retroperitoneal paraganglioma. Follow-up showed good recovery with no complications. Paraganglioma should be considered when an unknown mass with severe blood pressure fluctuations is encountered during surgery. Careful preoperative preparations and close intraoperative monitoring should be applied in patients with suspected paraganglioma.

## 1 Introduction

Pheochromocytomas and sympathetic paragangliomas (PPGLs) are rare neuroendocrine tumors, with 80%–85% originating in the adrenal medulla (pheochromocytomas) and 15%–20% in sympathetic paraganglia (paragangliomas) (1). Retroperitoneal paragangliomas, originating from extra-adrenal sympathetic ganglia cells, account for approximately 1–3% of retroperitoneal tumors. Epidemiological studies on PPGLs are limited, with no incidence or prevalence data reported in China. International studies suggested an incidence of 2–8 cases per million per year for pheochromocytoma (PCC), with 10%–20% occurring in children. The prevalence of PPGLs is 0.2%–0.6% in hypertensive patients and less than 0.05% in the general population (2). A recent nationwide study in the Netherlands reported age-standardized incidence rates of 0.46 for pheochromocytomas and 0.11 for paragangliomas per 100,000 person-years (3).

Patients with PPGLs can have excessive catecholamine produced from the tumor cells, leading to facial flushing, hypertension, palpitations, and anxiety. These clinical presentations could be atypical, which often leads to missed and misdiagnosis (4). An autopsy study reported a detection rate of undiagnosed PPGLs between 0.05%–0.1% (2). A median diagnostic delay was estimated to be approximately three years (5). Here, we present a patient with hypertensive crisis during the surgical removal of retroperitoneal mass that was finally confirmed as a paraganglioma by post-operative pathological study. We share our experience and lessens here in order to improve the healthcare to patients with similar conditions.

### 1.1 Clinical presentation

**Figure 1:**
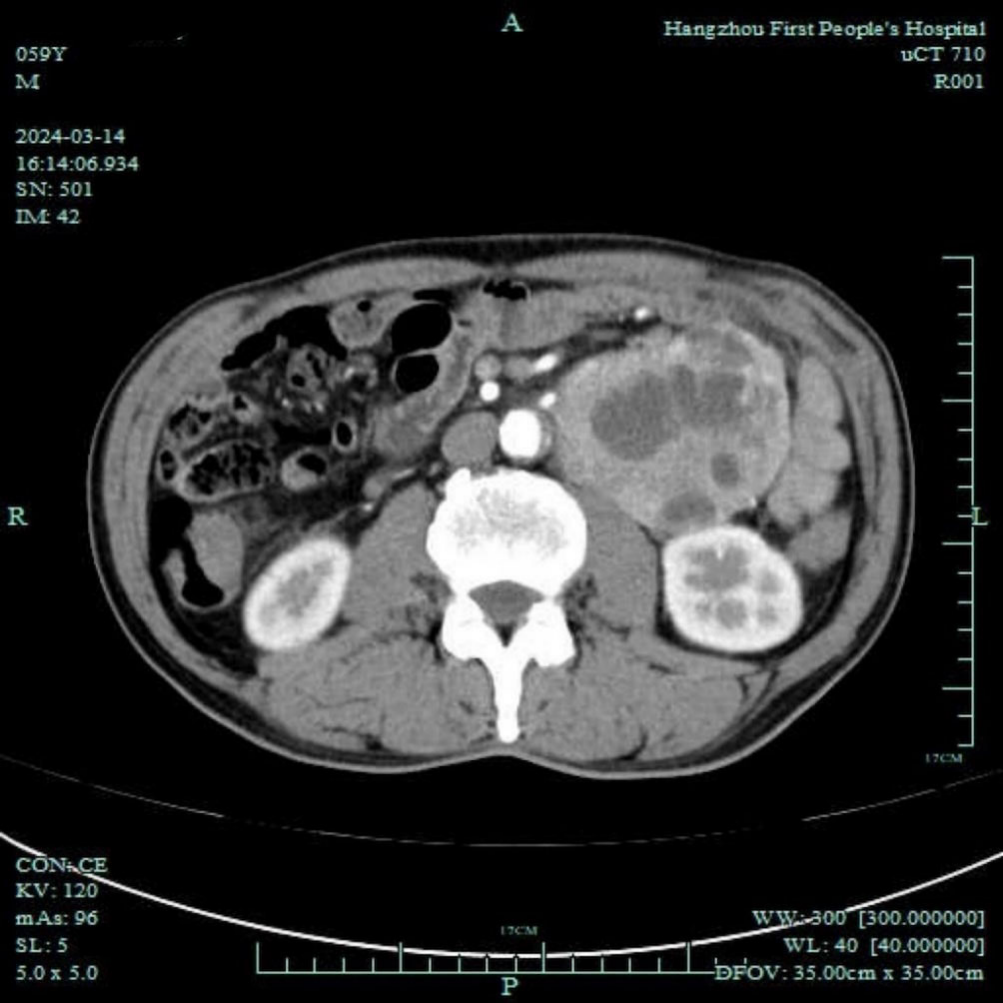
Abdominal enhanced computed tomography showing a lower-left abdominal tumor, approximately 91 × 70 mm in size, with indistinct boundaries adjacent to three segments of the small intestine, suggesting a malignant gastrointestinal stromal tumor or leiomyosarcoma.

The patient was a 60-year-old male, with height of 175 cm and weight of 62 kg. He was admitted to the endocrinology department due to unexplained weight loss of 16 kg over 7 years. The patient had a 7-year history of diabetes mellitus. Upon being admitted to the hospital, fasting blood glucose levels ranged from 12.0 to 13.2 mmol/L, with postprandial blood glucose peaking at 19.5 mmol/L. The patient also had a 7-year history of hypertension that required treatment with antihypertensive medications and beta-blockers. Through these medications, blood pressure control had been achieved and maintained effectively. Further abdominal ultrasound and enhanced computed tomography scan indicated a left lower abdominal tumor, approximately 91 × 70 mm in size, with indistinct boundaries adjacent to three small intestine segments, suggesting a malignant gastrointestinal stromal tumor or a leiomyosarcoma (**Figure 1**). The patient was transferred to the gastrointestinal surgery department for laparoscopic resection of the abdominal tumor under general anesthesia. His American Society of Anesthesiologists classification was II.

Preoperative oncology indicators are shown in **Table 1**, and preoperative ACTH and cortisol levels are shown in **Table 2**. Tests related to blood catecholamines or other pheochromocytomas and paragangliomas (PPGLs) were not conducted.

**Table 1:** Preoperative oncology indicators.

Test Item	Result	Reference range
AFP	5.33 μg/L	0.00–20.00 μg/L
NSE	16.80 μg/L	0.00–16.30 μg/L
CEA	6.04 μg/L	0.00–5.00 μg/L
CA19-9	30.55 μg/L	0.00–37.00 μg/L
CA125	9.5 μg/L	0.00–35.00 μg/L
CA15-3	17.36 μg/L	0.00–31.30 μg/L

AFP, Alpha-fetoprotein; NSE, Neuron-specific enolase; CEA, Carcinoembryonic antigen; CA19-9, Carbohydrate antigen 19-9; CA125, Cancer antigen 125; CA15-3, Cancer antigen 15-3.

**Table 2:** Preoperative ACTH and cortisol levels.

Test Item	Result	Reference range
ACTH 8 AM	28.00 ng/L	10.00–90.00 ng/L
ACTH 4 PM	22.00 ng/L	7.60–76.00 ng/L
Serum Cortisol (8 AM)	302.94 μg/L	43.00–224.00 μg/L
Serum Cortisol (4 PM)	106.74 μg/L	30.90–166.00 μg/L
24 h Urinary Cortisol	274.45 μg/24 h	28.50–213.70 μg/24 h

ACTH, Adrenocorticotropic Hormone.

Upon entering the operating room, the vital signs of the patient were heart rate 90 beats/min, blood pressure 137/74 mmHg, and oxygen saturation (SpO_2_) 98%. Radial artery cannulation and right internal jugular vein catheterization were performed under local anesthesia. Anesthesia induction was achieved with intravenous propofol (20 mg), sufentanil (20 μg), rocuronium (50 mg), and remimazolam (5 mg). Endotracheal intubation and mechanical ventilation were established.

At the initiation of the laparoscopic procedure, the vital signs of this patient remained stable, with an anesthesia depth of 50 (bispectral index). However, 37 minutes into the procedure, his blood pressure rose to 180/110 mmHg. Sufentanil 10 μg and urapidil 15 mg were administered intravenously, reducing the blood pressure to 120/75 mmHg. During the ongoing manipulation of the procedure, the blood pressure fluctuated, with the peak at 285/134 mmHg but returning to normal when the manipulation was halted. Continuous intravenous phentolamine infusion was initiated, but his blood pressure still fluctuated with each tumor manipulation.

### 1.2 Diagnosis and treatment

We then suspected a catecholamine-secreting paraganglioma. The laparoscopic procedure was converted to an open abdominal surgery. The phentolamine infusion was adjusted based on the blood pressure measurements. Venous blood samples were collected for analysis. Blood pressure spikes occurred repeatedly during surgery and were controlled effectively with phentolamine infusion. After the abdominal mass was resected at 130 minutes into the operation, his blood pressure suddenly dropped to 44/28 mmHg, with the heart rate increasing to 100–123 beats/min. Norepinephrine 1 mg was administered, followed by continuous infusion at 0.1–0.5 μg/kg/min to maintain the blood pressure at 98–140/60–80 mmHg. The surgery lasted a total of 365 minutes, requiring 2,500 mL of lactated Ringer’s solution and 1,000 mL of infused hydroxyethyl starch, with 150 mL of blood loss and 600 mL of urine output.

**Figure 2:**
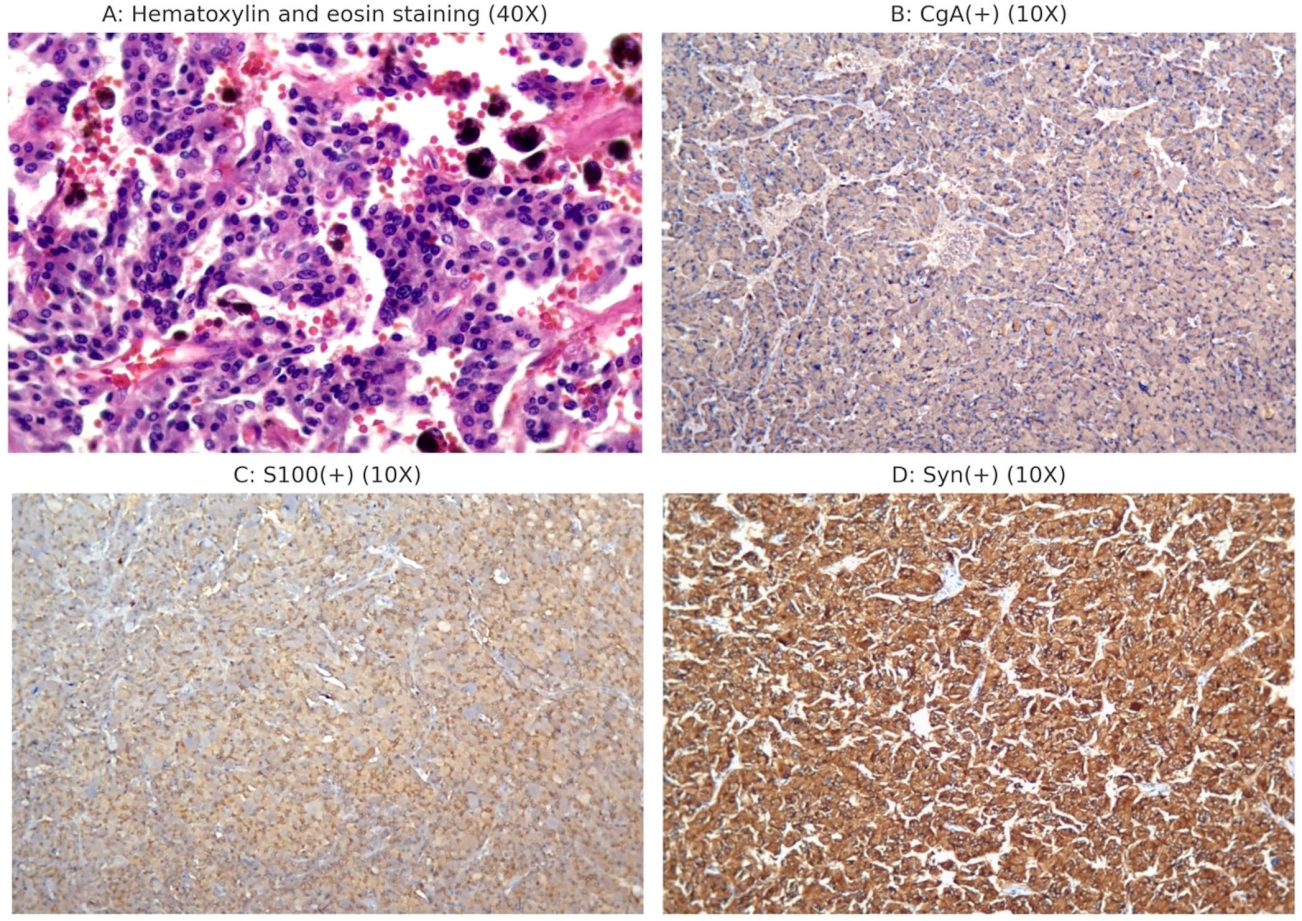
Postoperative histopathology and immunohistochemistry examination. **(A)** Postoperative pathology showed a patchy distribution of tumor cells with blue-purple cytoplasm and abundant interstitial blood sinuses. **(B)** CgA (+); **(C)** S100 (+); **(D)** Syn (+).

Postoperatively, the patient was transferred to the post-anesthesia care unit (PACU). Intravenous normal saline infusion was continued, with a norepinephrine drip at 0.05–0.2 μg/kg/min. After 20 minutes, the patient was extubated after he regained his muscle strength and consciousness. Norepinephrine was gradually discontinued 30 min later once the vital signs were stabilized. The patient was transferred back to the medical ward 1 hour later and discharged five days postoperatively. Postoperative pathology diagnosed the retroperitoneal tumor as PGL (**Figure 2**). **Table 3** presents the plasma catecholamines and their metabolites levels measured during and after surgery.

**Table 3:** Plasma catecholamines and their metabolites levels during and after surgery.

Indicator	Intraoperative result (pg/mL)	Postoperative result (pg/mL)	Reference range (pg/mL)
Dopamine	368	24.1	<20.00
Metanephrine	5290	34.6	<262.00
Norepinephrine	53500	613	217.00–1109.00
Normetanephrine	7970	222	<145.00
Epinephrine	11700	45.9	<95.00
3-methoxytyramine)	68.1	8.47	<18.40

## 2 Discussion

Pheochromocytomas are tumors of chromaffin cells in the adrenal medulla, usually producing the catecholamines, including epinephrine, norepinephrine, and dopamine. Paragangliomas are extra-adrenal chromaffin cell tumors arising from sympathetic paraganglia in the thorax, abdomen, and pelvis. These tumors may also originate from parasympathetic ganglia in the neck and skull base, such as the glossopharyngeal and vagus nerves, which do not produce catecholamines (6). Yang et al (7). previously reported a similar case in which an unexpected paraganglioma was discovered during the resection of a posterior mediastinal mass. During the surgery, severe hemodynamic changes occurred, and postoperative pathological examination revealed a rare functional paraganglioma in the posterior mediastinum.

In this case, the patient’s vital signs were stable from the start of surgery until the tumor was manipulated, which caused a sharp rise in blood pressure. Sedation, analgesia, and urapidil were ineffective in lowering blood pressure. Each tumor manipulation induced a significant hypertensive response. Thus, we suspected a catecholamine-secreting paraganglioma. Effective blood pressure control with phentolamine infusion supported this suspicion. Plasma-free normetanephrine and metanephrine levels and postoperative histopathology confirmed this as a rare abdominal paraganglioma (**Table 3**, **Figure 2**).

Reviewing the patient’s history revealed unexplained weight loss of 16 kg over the past 7 years, with severe constipation, poor glycemic control despite insulin therapy, and hypertension under three medication treatments. The medical history and auxiliary examinations were consistent with PPGL (1).

Adrenal masses typically prompt clinicians to conduct comprehensive biochemical, radiological, genetic, and cardiac evaluations preoperatively, with individualized treatment plans to reduce life-threatening intraoperative complications (8). In contrast, masses in the thoracic, abdominal, or pelvic cavities lack specific clinical symptoms except the triad of headaches, palpitations, and sweating (40%–48%) (2), making the diagnosis of paragangliomas difficult to detect and a great clinical challenge. Optimal preoperative PPGL assessments require multidisciplinary involvement, including comprehensive plasma catecholamines and their metabolites, imaging, cardiac, and genetic evaluations (9). In patients with suspected PPGL, proper preoperative assessments combined with strict blood pressure and heart rate control and optimized blood volume status can significantly reduce intraoperative and perioperative complications (10). The preoperative evaluation of pheochromocytoma requires multidisciplinary involvement, encompassing comprehensive blood catecholamine and metabolite testing, imaging studies, cardiac evaluations, and genetic assessments. For patients suspected of having pheochromocytoma, appropriate preoperative evaluation, combined with strict blood pressure and heart rate control, as well as optimization of blood volume status, is indispensable. All patients with PPGL require preoperative preparation beginning at least 10–14 days before surgery. Treatment should maintain blood pressure below 130/80 mmHg in the supine position while ensuring systolic pressure remains above 90 mmHg when upright. Phenoxybenzamine, a noncompetitive α-adrenoreceptor antagonist, remains the most widely administered adrenergic blocker. The standard regimen initiates with 10 mg twice daily, followed by dose titration as needed (8). In patients with tachycardia, β-blockers should be initiated only after achieving sufficient α-blockade to avoid precipitating a hypertensive crisis (9).

Anesthesiologists play a critical role in managing intraoperative complications, and with appropriate perioperative management strategies can reduce the associated mortality (9). In our patient, we did not suspect PPGL but considered a gastrointestinal stromal tumor or an interstitial malignant tumor based on the preoperative laboratory tests and image results. Without targeted PPGL testing (e.g., plasma-free metanephrines) and the subsequent preoperative preparations upon positive results, the patient experienced a severe intraoperative blood volume deficiency and hypertensive crises during tumor manipulation.

For patients suspected of having pheochromocytoma, invasive arterial blood pressure monitoring and central venous access are indispensable (11). Advanced hemodynamic monitoring is equally critical, as it facilitates the evaluation of blood volume and cardiac contractility through techniques such as Swan-Ganz catheter monitoring (12) or transesophageal echocardiography, thereby enabling early detection of cardiac complications during hypertensive crises (13).

In addition, it is important to note that during the removal of pheochromocytomas or paragangliomas, the blood catecholamine concentration can undergo significant changes. These changes are typically rapid and significant. The anesthesiologist should choose appropriate medications to mitigate these hemodynamic changes. The three most critical time points are during tracheal intubation, tumor manipulation, and tumor vessel ligation (14). During the first two periods, there is a massive release of catecholamines, which can result in hypertension and arrhythmias, as observed in this patient, whose blood pressure surged to a maximum of 285/134 mmHg during tumor manipulation. Conversely, during the latter period, the rapid decrease in blood catecholamine levels can cause a sharp drop in blood pressure, which was shown in this patient, with the blood pressure plummeted to a nadir of 44/28 mmHg after the tumor vessels were ligated.

It is crucial to maintain adequate blood volume from the start of surgery to prevent complications. Initially, physicians should supplement with crystalloids, then evaluate for hypovolemia using dynamic hemodynamic parameters (e.g., stroke volume or pulse pressure variations). If significant bleeding occurs, colloids or blood products should be used to replenish blood volume. If hypotension persists after fluid resuscitation, intravenous epinephrine or norepinephrine should be administered to restore normal blood pressure (9). In cases of refractory hypotension, vasopressin may be used, as its action is independent of adrenergic receptors (15).

Most patients do not require intensive care unit admission postoperatively but should be transferred to the PACU (13). The heart rate, blood pressure, and blood glucose should be closely monitored for 24–48 hours (8). This patient was able to return to the ward after 1-hour stay at PACU, due to our timely PPGL identification, adequate volume expansion, and appropriate vasoactive medication (phentolamine for blood pressure control during tumor resection and norepinephrine for blood pressure support post-resection). Kang et al (16). reported two cases where the nature of retroperitoneal masses was unclear before surgery. Preoperative imaging suggested possible diagnosis of “small-bowel gastrointestinal stromal tumor (GIST)” and “leiomyosarcoma”. During the surgical procedure, manipulation of the masses by the surgeon resulted in elevated blood pressure and increased heart rate. Multiple administrations of nicardipine and esmolol failed to control blood pressure and heart rate. Due to the unavoidable need for repeated manipulation of the masses, one of the surgeries had to be aborted. Surgical resection of paraganglioma should ideally occur within 25 hours of detecting an undiagnosed mass accompanied by severe, labile hypertension, given the urgency of managing catecholamine-secreting tumors.

The patient was first admitted to the endocrinology department due to weight loss that was initially thought to be caused by poor management of blood sugar control of type 2 diabetes. Hence, examinations were conducted around type 2 diabetes. Upon examination an abdominal mass was found, which the radiology department considered stromal tumor, and the patient was transferred to the gastrointestinal surgery department. The possibility of retroperitoneal paraganglioma was not considered in either department. Ultimately, perioperative management of PPGL is a major challenge for anesthesiologists and clinicians should increase the awareness of this disease to better diagnose and treat it.

## 3 Conclusions

In conclusion, PPGLs are rare illnesses. The clinical atypical presentations often lead to missed and misdiagnosed preoperatively. Surgery resection is the best treatment. However, patients with PPGLs can experience significant perioperative risks, with hypertensive crisis, especially during the manipulations of the tumor. The hypertensive crises induced by catecholamines in patients with PPGLs typically require multidisciplinary management. However, anesthesiologists are often the sole responders during intraoperative crises. Therefore, surgeons and anesthesiologists should improve their awareness of PPGLs, thoroughly review patient history and preoperative examination results, and, if necessary, organize multidisciplinary consultations. Clinicians should also consider paraganglioma when there is an unidentified mass is present.

## Data Availability

The original contributions presented in the study are included in the article/supplementary material. Further inquiries can be directed to the corresponding author/s.
